# The Inhibitory Effect of Curcumin on Virus-Induced Cytokine Storm and Its Potential Use in the Associated Severe Pneumonia

**DOI:** 10.3389/fcell.2020.00479

**Published:** 2020-06-12

**Authors:** Ziteng Liu, Ying Ying

**Affiliations:** ^1^Jiangxi Province Key Laboratory of Tumor Pathogens and Molecular Pathology, School of Basic Medical Sciences, Nanchang University, Nanchang, China; ^2^Nanchang Joint Program, Queen Mary School, Nanchang University, Nanchang, China; ^3^Department of Pathophysiology, School of Basic Medical Sciences, Nanchang University, Nanchang, China

**Keywords:** curcumin, coronavirus, cytokine storm, pneumonia, lung injury

## Abstract

Coronavirus infection, including SARS-CoV, MERS-CoV, and SARS-CoV2, causes daunting diseases that can be fatal because of lung failure and systemic cytokine storm. The development of coronavirus-evoked pneumonia is associated with excessive inflammatory responses in the lung, known as “cytokine storms,” which results in pulmonary edema, atelectasis, and acute lung injury (ALI) or fatal acute respiratory distress syndrome (ARDS). No drugs are available to suppress overly immune response-mediated lung injury effectively. In light of the low toxicity and its antioxidant, anti-inflammatory, and antiviral activity, it is plausible to speculate that curcumin could be used as a therapeutic drug for viral pneumonia and ALI/ARDS. Therefore, in this review, we summarize the mounting evidence obtained from preclinical studies using animal models of lethal pneumonia where curcumin exerts protective effects by regulating the expression of both pro- and anti-inflammatory factors such as IL-6, IL-8, IL-10, and COX-2, promoting the apoptosis of PMN cells, and scavenging the reactive oxygen species (ROS), which exacerbates the inflammatory response. These studies provide a rationale that curcumin can be used as a therapeutic agent against pneumonia and ALI/ARDS in humans resulting from coronaviral infection.

## Introduction

During the Spanish influenza pandemic in 1917–1918, it was found that the deaths were not just seen in the elderly with weak immunity, but also young individuals with normal immunity. As part of a robust immune response in severe cases, the virus triggers overaction of immune systems, producing a large number of inflammatory factors, which causes severe damage to the lung and manifests acute respiratory distress syndrome (ARDS), resulting in high mortality. The same damaging effects of immune over-reaction were observed in outbreaks of severe acute respiratory syndrome coronavirus (SARS-CoV) (Huang et al., 2005; Channappanavar and Perlman, 2017), middle east respiratory syndrome CoV (MERS-CoV) (Channappanavar and Perlman, 2017), highly pathogenic avian influenza viruses (including H5N1 and H7N9) (Kalil and Thomas, 2019), and novel coronavirus (SARS-CoV2) (Yao et al., 2020).

Inflammation under physiological conditions is a protective mechanism that acts to eliminate exogenous agents invading to living bodies, remove necrotic tissues and cells, and promote damage repair (Netea et al., 2017). Being said that the inflammation initiates a protective immune response when it is confined to locally affected tissues. However, when the negative regulatory mechanism is suppressed, a persistent and extensive inflammatory reaction occurs, which can reach pathological levels causing fatally systemic damage (Torres et al., 2017). Such an inflammatory response, including overproduction of immune cells and pro-inflammatory cytokines, is defined as the cytokine storm that usually occurs in viral infection and causes acute lung injury (ALI) and ARDS. Resulting symptoms include congestion, atelectasis, and pulmonary edema, which affects oxygen exchange in the lung and eventually lead to death (Wheeler and Bernard, 2007). There is no effective regime for cytokine storm and resultant lung injury. Therefore, drugs to suppress the cytokine storm are urgently needed to treat deadly virus infection that causes lung damage and ARDS.

Curcumin[(1E,6E)-1,7bis(4-hydroxy-3-methoxyphenyl)-1,6-heptadiene-3,5-dione] is a natural medicine mainly extracted from plants of the Curcuma longa that has a long history to be used in humans in treating diseases without overt side effects. Numerous *in vitro* and *in vivo* studies indicate that curcumin has antioxidant, anti-inflammatory, anti-cancer, and anti-diabetic activity (Xu et al., 2018). Several clinical investigations have reported beneficial effects in treating cardiovascular diseases, metabolic syndrome, or diabetes, and infectious diseases, especially viral infection (Yang et al., 2014; Basu et al., 2013; Amalraj et al., 2017; Alizadeh et al., 2018; Asadi et al., 2019). All of these clinical findings point to that curcumin alleviates these diseases mainly via modulation of immune responses. Indeed, some preclinical studies have suggested that curcumin could inhibit the cytokine storm induced by the viral infection (Dai et al., 2018; Richart et al., 2018; Praditya et al., 2019; Vitali et al., 2020). Therefore, in this review, we outline the relationship between virus infections and cytokine storm and discuss the potential use of curcumin in treating viral infection-triggered ARDS. We hope to provide useful information and references for clinicians in combating devastating severe pneumonia caused by SARS-CoV2, a current global pandemic.

## Viral Infection and Cytokine Storm

Cytokine storm arises from different factors that could derive from autoimmune, inflammatory, iatrogenic, and infectious origins (Behrens and Koretzky, 2017). It is characterized by the production of excessive amounts of inflammatory cytokines as a result of unchecked feedforward activation and amplification of immune cells. Its clinical manifestations include systemic inflammation, multi-organ failure, hyperferritinemia, which is referred to as “cytokine storm syndrome” and could be lethal if untreated.

Under physiological conditions, the steady-state cytokine levels are maintained by negative and positive feedback regulation of their expression (Behrens and Koretzky, 2017). A large amount of virus in the body will induce over-reacted innate and adaptive immune response, triggering extravagant cytokines release and lymphocytes activation. Common to cytokine storm syndromes engendered by all insults is a loss of negative regulation of the production of inflammatory cytokines, which in turn drives a positive feedback regulation, leading to exponentially growing inflammation and multi-organ failure.

At an early stage, virus infection induces host cells to generate cytokines and chemokines, inflammatory mediators, and apoptosis of the host cells, which then attracts immune cells to the damaged areas (Liu et al., 2016). Macrophages, dendritic cells, and mast cells engulf antigen fragments, virus, and virus-bearing damaged cells, which triggers the production of the inflammatory mediators. Myeloid cells, including monocytes, neutrophils, and dendritic cells, contain multiple pattern recognition receptors (PRRs) on their surfaces to help them recognize and bind to viruses via Pathogen-associated molecular patterns (PAMPs) such as viral RNA/DNA, or damage-associated molecular patterns (DAMPs) from necrotic tissue and cells in aseptic inflammation. Subsequently, the immune cells are activated and produce pro-inflammatory cytokines, including tumor necrosis factor-α (TNF-α), interleukin (e.g., IL-1β, IL-6), and interferon-gamma (IFN-γ) (Taniguchi and Karin, 2018). The release of cytokine causes increased vascular permeability; consequently, the leukocytes increasingly migrate to damaged tissues through margination, rolling, adhesion, transmigration, and chemotaxis. Activated leukocytes simultaneously release prostaglandins and inflammatory factors, and activate the complement system, producing C3a and C5a components that kill pathogens (Medzhitov, 2008; Straub et al., 2015; Netea et al., 2017).

An additional effect of cytokines is to activate NADPH oxidase in leukocytes, leading to the generation of reactive oxygen species (ROS) such as superoxide, hydroxyl radicals, and singlet oxygen (Liu et al., 2016). On the one hand, ROS helps to remove proteins, lipids, and nuclear acids of the damaged cells and activate immune cells to eliminate foreign microorganisms through extracellular mechanisms (Zhang et al., 2016). On the other hand, ROS acts as a second messenger to regulate intracellular signaling events. For example, it activates the nuclear factor-κB (NF-κB) to promote further production of pro-inflammatory cytokines such as TNF-α, IL-6, IL-8, and other inflammatory factors (Baldwin, 1996; Cohen et al., 2009; Zhang et al., 2016; Hellebrekers et al., 2018; Khan and Khan, 2018). Therefore, pro-inflammatory cytokines and ROS exert forward feedback regulation of their production.

**FIGURE 1:**
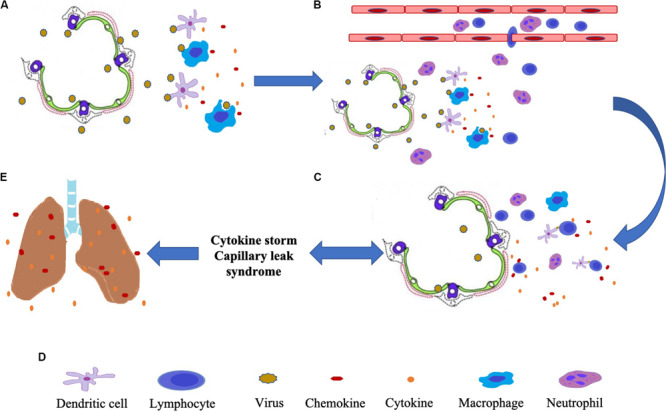
The diagram of lung injury caused by virus-induced cytokine storms. **(A)** The viruses attack alveolar epithelial cells and are recognized by dendritic cells and macrophages, which then release cytokines. **(B)** Cytokines and chemokines help white blood cells in the blood reach the alveoli. **(C)** Antigen-presenting cells (dendritic cells) activate lymphocytes. Activated lymphocytes produce and release large amounts of cytokines while attacking infected alveolar epithelial cells. **(D)** Induce cytokine storm, and capillary leak syndrome. **(E)** Causes atelectasis, pulmonary edema, pulmonary congestion, and ARDS.

The inflammatory response can be turned off by the anti-inflammatory cytokine IL-10 (Opal and DePalo, 2000). The positive and negative regulatory inputs maintain normal innate immunity. However, if the balance is disturbed in some cases, for instance, inhibition of the immuo-suppressor cytokine IL-10, a cytokine storm takes place. Infections from such viruses as Ebola, avian influenza, dengue, and coronavirus, can lead to cytokine storms, producing a massive amount of pro-inflammatory cytokines. The concerted action of these inflammatory mediators causes the destruction of tissues and cells, manifested by clinical syndromes such as extensive pulmonary edema, alveolar hemorrhage, ARDS, and multiple organ failures (Matthay and Zimmerman, 2005; Lau et al., 2013; Sordillo and Helson, 2015; Channappanavar and Perlman, 2017; Amini et al., 2018) (Figure 1).

There is clear evidence from coronavirus infected patients with both high cytokine levels and pathological changes in the lung (Wang et al., 2007; Channappanavar et al., 2016; Chen et al., 2020; Wu et al., 2020). For example, in plasma of COVID-19 patients, high concentrations of IL-2, IL-6, and IL-7 have been observed (Chen et al., 2020; Green, 2020; McGonagle et al., 2020; Wu et al., 2020). In particular, IL-6 was significantly elevated in critically ill patients with ARDS compared to patients without ARDS and was statistically significantly correlated with death (Wu et al., 2020). Both patients with mild or severe symptoms had pneumonia, and 29% of patients developed ARDS (Wang et al., 2020).

## Curcumin Inhibits Inflammatory Reaction

### Inhibition of the Production of Pro-Inflammatory Cytokine

Numerous *in vivo* and *in vitro* studies have been shown that curcumin and its analogs markedly inhibit the production and release of pro-inflammatory cytokines, such as IL-1, IL-6, IL-8, TNF-α (Avasarala et al., 2013; Zhang et al., 2015; Dai et al., 2018; Zhang et al., 2019). In line with this, Zhang et al. (2019) have observed that direct pulmonary delivery of solubilized curcumin dramatically diminishes pro-inflammatory cytokines IL-1β, IL-6, TNF-α in the BAL cells, the lung and serum of mice with severe pneumonia induced by Klebsiella. In addition, curcumin also decreases expression of many other inflammatory mediators, including MCP1(CCL2), MIPI1 (CCL3), GROα (CXCL1), GROβ (CXCL2), IP10 (CXCL10), SDF1 (CXCL12), MMP-2, IFN-γ, and MMP-9, which regulate the activity of immune cells and inflammatory responses and promote fibrosis in the lung after infection (Sordillo and Helson, 2015; Dai et al., 2018).

**FIGURE 2:**
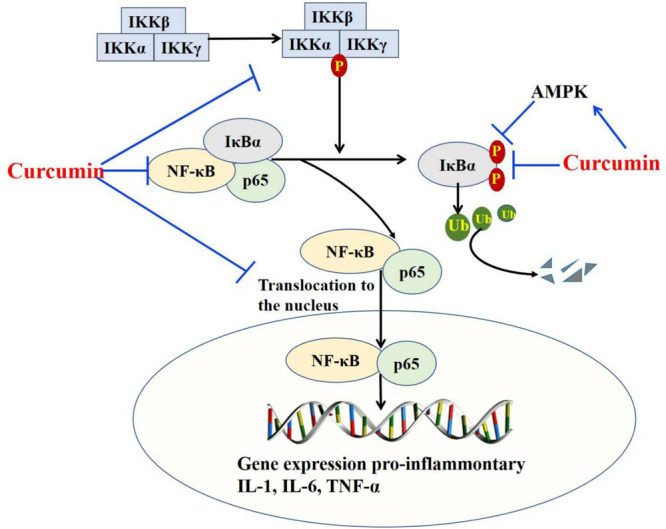
Curcumin inhibits the production of pro-inflammatory cytokine by targeting the NF-κB pathway. Curcumin targets NF-κB signaling through inhibiting activation of IKKβ, enhancing expression or stability of IκBα, activating AMPK, and targeting P65.

The mechanism underlying curcumin modulation of inflammation has been extensively investigated and engages diverse signaling pathways, among which NF-κB plays an essential role (Cohen et al., 2009; Salminen et al., 2011; Han et al., 2018). It was reported that curcumin effectively regulates NF-κB signaling through multiple mechanisms (Figure 2): First, curcumin inhibits activation of IKKβ (Cohen et al., 2009). In a study of patients with head and neck cancer receiving curcumin, reduced activity of IKKβ was observed in saliva samples, associated with a decrease in the expression of IL-8, TNF-α, and IFN-γ (Kim et al., 2011). Second, curcumin enhances the expression or stability of IκBα (Jobin et al., 1999; Han et al., 2018; Chen et al., 2019). Curcumin inhibits the IκBα degradation, phosphorylation of IκB serine 32 to block the cytokine-mediated NF-κB activation and thus pro-inflammatory gene expression (Jobin et al., 1999). Third, curcumin activates AMPK (Han et al., 2018). It has been documented that curcumin blocks NF-κB signaling upon infection with Influenza A virus (IAV) as a consequence of AMPK activation (Han et al., 2018). Fourth, curcumin acts on p65 to disturb the NF-κB pathway (Xu and Liu, 2017). Infection with IAV led to a decrease of p65 in the cytosol of macrophages and a corresponding increase in the nucleus, where it forms a functional complex with NF-κB, ultimately upregulating transcription of pro-inflammatory cytokines. In contrast, the use of curcumin blocks the nuclear translocation of NF-κB and p65, downregulating transcription of the cytokine genes (Xu and Liu, 2017).

Other inflammatory mediators have been reported to be regulated by curcumin. One of them is cyclooxygenase 2 (COX-2), a key enzyme for the synthesis of prostaglandin (Khan and Khan, 2018). In an animal model of chronic obstructive pulmonary disease, it has been shown that curcumin treatment effectively inhibits the degradation of IκBα and disturbs the production of COX-2 (Yuan et al., 2018). In addition to disrupting the NF-κB pathway, curcumin inhibits the virus-induced expression of TLR2/4/7, MyD88, TRIF, and TRAF6 genes, and blocks IAV-induced phosphorylation of Akt, p38, JNK as well (Sordillo and Helson, 2015; Dai et al., 2018).

### Regulation of Anti-inflammatory Cytokines

In contrast to its negative effect on pro-inflammatory molecules, curcumin has been shown to regulate anti-inflammatory cytokines positively, in particular IL-10 (Larmonier et al., 2008; Chen et al., 2018; Mollazadeh et al., 2019; Chai et al., 2020). The latter is an essential negative regulator for inflammatory responses and is secreted by the dendritic cells that bind to DAMP released from damaged cells during aseptic or antigenic inflammatory reactions. IL-10 acts on inflammatory monocytes to reduce the release of TNF-α, IL-6, and ROS, thereby alleviating tissue damage caused by the continuous inflammatory response (Bamboat et al., 2010). Moreover, IL-10 drives the differentiation of Tregs (Mollazadeh et al., 2019). An early study has shown that IL-10 reduces the expression of intercellular adhesion molecule-1 (ICAM-1) on pulmonary vascular and TNF-α levels, which cause reduction of the expression of myeloperoxidase and the number of neutrophils in BAL fluids, consequently alleviating the lung damage (Mulligan et al., 1993).

Many studies have revealed that curcumin and curcuminoids potently increase the expression, production, and activity of IL-10 (Larmonier et al., 2008; Chen et al., 2018; Mollazadeh et al., 2019; Chai et al., 2020). Chai et al. (2020) have depicted the effect of curcumin on ALI/ARDS using cecal ligation and puncture (CLP)-induced ALI mouse model. In this study, curcumin noticeably attenuates lung injury by inducing the differentiation of regulatory T cells (Tregs) and upregulating IL-10 production. Similar effects have been observed in the neuropathic model, colitis model, and other inflammatory diseases. Therefore, in the context of inflammation, curcumin can act as a double-edged sword, downregulating pro-inflammatory cytokines, and upregulating anti-inflammatory IL-10 (Chai et al., 2020).

## Scavenges ROS

It has been described that curcumin acts to directly scavenge ROS as a polyphenolic antioxidant (Wang et al., 2008). Curcumin has two active groups, one hydroxy hydrogen on the benzene ring that has an anti-oxidation effect and the other a β-diketone moiety. *In vitro* experiments have shown that curcumin effectively scavenges on ROS removal and anti-oxidation, curcumin has been shown effective at scavenging the superoxide anion radical produced by illuminating riboflavin and the OH^–^ produced by the Fenton reaction. Curcumin also inhibits the peroxidation of lecithin and DNA oxidative damage caused by ROS (Wang et al., 2008).

The ability of curcumin to scavenge ROS can be indirect via enzymatic regulation. For example, curcumin can upregulate superoxide dismutase 2 (SOD2), a key enzyme to convert O^2–^ to H_2_O_2_, which is then reduced to H_2_O by glutathione (GSH) redox system (Forrester et al., 2018). In a study examining liver damage in rats, the GSH redox system was shown to be inhibited by the folic acid antagonist Methotrexate, resulting in hepatic oxidative damage. Curcumin is able to reverse this effect and enhance the effectiveness of SOD so as to maintain the oxidant/antioxidant balance and mitigate liver damage (Hemeida and Mohafez, 2008). Recently, curcumin was reported to oppose the effect of ROS on pro-inflammatory cytokine expression (e.g., IL-1b, IL-18) by downregulation of the thioredoxin interacting protein/NLR pyrin domain containing 3 (TXNIP/NLRP3) (Ren et al., 2019).

## Antiviral Activity of Curcumin

Many studies have documented that curcumin disrupts the viral infection process via multiple mechanisms, including directly targeting viral proteins, inhibiting particle production and gene expression, and blocking the virus entry, replication, and budding (Wen et al., 2007; Basu et al., 2013; Ou et al., 2013; Du et al., 2017; Kannan and Kolandaivel, 2017; Yang et al., 2017; Dai et al., 2018; Praditya et al., 2019). A recently *in vitro* study has demonstrated that curcumin inhibits respiratory syndrome virus (RSV) by blocking attachment to host cells (Yang et al., 2017). In this study, curcumin was also found to prevent the replication of RSV in human nasal epithelial cells. Additional evidence suggests that curcumin inhibits Porcine reproductive and RSV (PRRSV) attachment, possibly by disrupting the fluidity of viral envelopes (Du et al., 2017). Curcumin also obstructs virus infection by inhibiting PRRSV-mediated cell fusion, virus internalization, and uncoating (Du et al., 2017).

For a century, different subtypes of IAV, H1N1, H2N2, H3N2, and H5N1 have been the leading cause of pandemic outbreaks in the world. It has been reported that curcumin and its derivatives have a high binding affinity to hemagglutinin (HA), a major capsid glycoprotein of influenza virus that mediates virus attachment (Kannan and Kolandaivel, 2017). Ou et al. (2013) have demonstrated that curcumin interacts with HA and disturbs the integrity of membrane structure to block virus binding to host cells and prevent IAV entry. In another study with cells infected by IAV, it was found that curcumin directly inactivates various strains of IAV, disturbs their adsorption, and inhibits their replication (Dai et al., 2018). Further, the study showed that curcumin inhibits IAV absorption and replication by activating the NF-E2-related factor 2 (Nrf2)-hemeoxygenase-1 (HO-1)-axis, a classical anti-inflammatory and antioxidative signaling, which possesses antiviral activity (Dai et al., 2018).

Furthermore, curcumin acts against SARS-CoV (Wen et al., 2007). Accordingly, a study on the anti-SARS-CoV activity of 221 phytocompounds revealed that 20 μM of curcumin exhibits significant inhibitory effects in a Vero E6 cell-based cytopathogenic effect (CPE) assay. The authors presented evidence for a mild effect of curcumin against SARS-CoV replication and the inhibitory effect of curcumin on SARS-CoV 3CL protease activity, which is essential for the replication of SARS-CoV. This study provides promising evidence for curcumin as a potential anti-SARS-CoV agent (Wen et al., 2007).

## Curcumin Alleviates Exudation and Edema Caused by Inflammation

Inflammation plays a pivotal role in the pathogenesis of lung complications of viral infection, as manifested by lung edema, hemorrhage, neutrophil infiltration, and alveolar thickening. Studies indicate that curcumin and its analogs are capable of attenuating lung injury (Suresh et al., 2012; Avasarala et al., 2013; Zhang et al., 2015; Xiao et al., 2019). Polymorphonuclear neutrophils (PMNs) infiltration is associated with pulmonary edema and could release oxidants and proteases, which consequently damage the alveolar-capillary membrane, leading to leakage of plasma proteins out of blood vessels, thereby causing pulmonary edema (Matthay and Zimmerman, 2005). It has been shown that curcumin can inhibit the infiltration of PMNs, including (GR1^+^), CD4^+^, CD19^+^ B cells, NK cells, and CD8^+^ T cells, and promote the apoptosis of PMN by increasing the level of P-p38 (Avasarala et al., 2013). More recently, Xiao et al. (2019) have reported that curcumin analog C66 protects lipopolysaccharide (LPS)-induced ALI through suppression of the JNK pathway and subsequent inhibition of inflammatory cytokine expression. Similar protective effects of curcumin have been reported in the rodent model of ventilator-induced lung injury (Wang et al., 2018) and Staphylococcus *S.aureus*-induced ALI (Xu et al., 2015) as evidenced by attenuation of inflammatory cell infiltration, lung edema due to its anti-inflammatory and antioxidant effects. In a chronic obstructive pulmonary disease model, curcumin treatment effectively reduces the degree of airway inflammation and disrupts airway remodeling by inhibiting the proliferation of bronchial epithelial cells (Yuan et al., 2018).

Mechanistically, curcumin protects the lung by inhibiting inflammation and production of ROS through regulation of multiple signaling pathways engaging peroxisome proliferator-activated receptor γ (PPARγ) (Cheng et al., 2018), JNK (Xiao et al., 2019), NF-κB (Suresh et al., 2012; Wang et al., 2018), and Nrf2 (Dai et al., 2018; Han et al., 2018). Notably, the role of curcumin in regulating Nrf2/HO-1 has been reported in IAV infection (Dai et al., 2018; Han et al., 2018). The Nrf2 enhances the expression of HO-1, an immunoregulatory and anti-inflammation molecule, and other enzymes for maintaining redox homeostasis. The increased expression of HO-1 can alleviate the pathological remolding of the lung during viral infection and increase the survival rate in mice following IAV infection. Curcumin has been shown to stimulate transcription of Nrf2 and thus enhance HO-1 expression *in vivo*, protecting alveoli from merging, inflating and enlarging, and decreasing inflammatory exudation of proteins to alveoli spaces after infection (Dai et al., 2018; Han et al., 2018).

## Curcumin Suppresses Fibrosis

The ALI after the viral infection is often followed by pulmonary fibrosis, which can lead to the death. It has been reported that curcumin can inhibit pulmonary fibrosis. Thus, in paraquat-treated mice, collagen deposition in the lung causes diffused fibrosis, while treatment with curcumin reduces collagen deposition and decelerates the development of pulmonary fibrosis (Chen et al., 2017). In the radiation-induced lung damage model, cytokine accumulation and collagen deposition occur in the interstitial space, concurrent with fibrosis of the lung tissue (Amini et al., 2018). However, curcumin reduces the expression of cytokines such as IL-4 and TGF-β, inhibits the infiltration of macrophages and lymphocytes, and ameliorates fibrosis (Amini et al., 2018). In another study on ALI using a mouse model infected with reovirus, curcumin treatment effectively inhibits the production of collagen and procollagen I mRNA (Avasarala et al., 2013). α-SMA, a marker of epithelial to mesenchymal transition, and Tenascin-C (TN-C), both of which are indicators of pulmonary fibrosis, are highly expressed in the adult lung parenchyma after ALI. The high expression of E-cadherin, accompanied by cell proliferation and repair, is associated with pulmonary remodeling after lung injury. Treatment with curcumin reduces the expression of TN-C, α-SMA, and E-cadherin attenuates myofibroblast differentiation and mitigates pulmonary fibrosis. Furthermore, curcumin decreases the expression of the TGF-β receptor II (TGF-ß RII), suggesting that it prevents TGF-β-mediated pulmonary fibrosis. In bleomycin/SiO2/amiodarone-induced pulmonary fibrosis experiments, it was also demonstrated that curcumin directly reduces the expression of TGF-β protein and its mRNA (Avasarala et al., 2013). All these studies support that curcumin alleviates pulmonary fibrosis.

## The Potential Role of Curcumin in the Prevention and Treatment of Coronavirus Infection

In the last two decades, coronavirus infection has gained much attention for its high mortality. The consensus from recent research is that the cytokine storm plays a crucial role in the development and progression of fatal pneumonia. Among those who experienced SARS-CoV infection in 2003, many manifested ALI and developed ARDS, and the death rate was greater than 10% (Peiris et al., 2004). Similar syndromes are seen in the MERS-CoV, H5N1, H7N9, and SARS-CoV2 infection. The high mortality rate from fatal pneumonia is due to the over-activation of immune cells in the lung (Channappanavar and Perlman, 2017).

Targeting cytokine storm is considered as an essential strategy for CoV infections. In clinical settings, glucocorticoids have been used to treat fatal viral pneumonia and shown therapeutic benefits. In the treatment of patients with SARS in 2003, glucocorticoids were widely used to suppress the cytokine storm in severe cases (Buchman, 2001). However, it has been found that large doses of glucocorticoids create many side effects such as osteoporosis and secondary infection with other pathogenic microbes, and small doses have little effect on improving lung injury (Buchman, 2001). These clinical findings indicate that it is increasingly important to seek alternative agents with effectiveness and low toxicity.

Many studies on virus-induced pneumonia have highlighted the potential usage of curcumin in the improvement of lung index and survival rate (Avasarala et al., 2013; Xu and Liu, 2017; Dai et al., 2018; Han et al., 2018; Lai et al., 2020). Curcumin mitigates the severity of viral pneumonia through inhibiting the production of inflammatory cytokines and NF-κB signaling in macrophages (Xu and Liu, 2017; Han et al., 2018). Curcumin has also been shown to activate Nrf2 in association with reduced oxidative stress and inhibit TLR2/4, p38/JNK MAPK, and NF-κB in response to IAV infection; and as a result, pneumonia is improved (Dai et al., 2018).

Up to now, it has been claimed that curcumin benefits human health and prevents diseases (DiSilvestro et al., 2012; Zhu et al., 2019). A recent study suggested that a low dose of curcumin (80 mg/day) produced a variety of health-promoting actions, such as direct and indirect antioxidant actions (DiSilvestro et al., 2012). Additionally, accumulating evidence from animal studies has shown that curcumin prevents the development of severe pneumonia. Thus, pre-treatment of curcumin (5 mg/kg/day) inhibits paraquat-induced lung inflammation and structural remodeling of the lung at an early phase of ALI (Tyagi et al., 2016). Bansal and Chhibber (2010) have demonstrated that pre-treatment of mice with curcumin (150 mg/kg) for 15 days before Klebsiella *pneumonia* infection prevents the tissue from injury and reduces ALI-associated pneumonia by the anti-inflammatory action of curcumin. The similar protective role of curcumin has been found in preclinical studies of viral-induced pneumonia. Treatment with curcumin (50 mg/kg/day) beginning at 5 days prior to reovirus 1/L infection protects CBA/J mice from the development of ALI/ARDS and suppresses subsequent fibrosis (Avasarala et al., 2013). Lai et al. (2020) have reported that pre-infection or post-infection administration of curcumin significantly improves the lung index and prolongs the survival rate. Interestingly, the fatality rate is also reduced by pre-administration with curcumin (Lai et al., 2020). All these studies suggest that curcumin administration could have both prophylactic and therapeutic effects on virus-induced pneumonia and mortality.

Clinical investigations have suggested that curcumin might be effective in improving inflammation and the treatment of virus infections. A clinical trial conducted by Alizadeh et al. (2018) have demonstrated that curcumin nanomicelle supplement ameliorates oxidative stress, and reduces inflammatory biomarker, including TNF-α, compared to a placebo. Furthermore, a phase II randomized controlled study has reported that the topical application of curcumin and curcumin polyherbal cream has a higher HPV clearance rate than the placebo (Basu et al., 2013).

Currently, no data in humans on the link between curcumin and coronavirus infection have been available, but in light of and its preventative and therapeutic role in viral infection and cytokine storms common to all viral infections, curcumin could conceivably be considered as an attractive agent for the management of coronavirus infections.

## Conclusion

**FIGURE 3:**
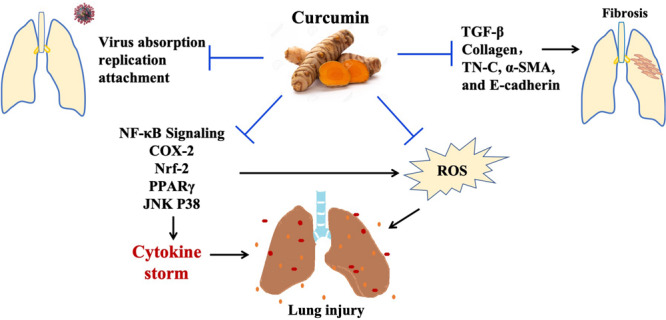
The effects of curcumin in virus associated with severe pneumonia. Curcumin inhibits virus-induced lung injury through its antivirus, anti-inflammation, antioxidant activity. In addition, curcumin could suppress fibrosis by targeting TGF-β signaling. Abbreviations: Nrf2, nuclear factor erythroid-derived 2; NF-κB, nuclear factor-κB; PPARγ, peroxisome proliferator-activated receptor γ; TNF-α, tumor necrosis factor-alpha; COX2, cyclooxygenase-2; IκB, inhibitor of kappa B; IL, interleukin; JNK, c-Jun N-terminal Kinase; TN-C, tenascin-C; α-SMA, alpha-smooth muscle actin.

Cytokine storm syndrome triggered by viral infections is the culprit of death. It is exacerbated by unchecked regulation of the production of pro-inflammatory cytokines and ROS, leading to pneumonia, ALI, multiple organs failures, and eventually death. No effective therapy is available for the cytokine storm syndrome and associated lung and other organ failures. Curcumin is a natural plant extract with high safety and low toxicity such that people take it as a diet supplement, and growing evidence from preclinical studies demonstrates that it effectively inhibits viral infection, alleviates the severity of lung injury through offsetting the cytokine storm, inhibits subsequent fibrosis, and increases survival rates (Figure 3). Additionally, its anti-SARS-CoV replication and 3CL protease have been reported in an *in vitro* study (Wen et al., 2007). In sum, the preclinical studies we have reviewed here motivate a call for attention to the clinical investigation of curcumin as a therapeutic agent for the cytokine storm syndrome following coronavirus infections, especially pneumonia caused by the coronavirus.

## Author Contributions

ZL contributed to the preparation of the manuscript and editing. YY contributed to the literature research, revision, and final approval of the manuscript.

## Conflict of Interest

The authors declare that the research was conducted in the absence of any commercial or financial relationships that could be construed as a potential conflict of interest.
